# Synthesis of Sandwich-Structured Zeolite Molecular Sieves and Their Adsorption Performance for Volatile Hydrocarbons

**DOI:** 10.3390/ma18081758

**Published:** 2025-04-11

**Authors:** Tongyuan Liu, Wenxing Qi, Lihong Nie, Beifu Wang

**Affiliations:** School of Petrochemical and Environment, Zhejiang Ocean University, Zhoushan 316000, China; 18253763691@163.com (T.L.); 18115102970@163.com (W.Q.); nielihong@zjou.edu.cn (L.N.)

**Keywords:** pore engineering, monolithic zeolite, ZSM-5, VOCs, sandwich structure

## Abstract

To address the issue of volatile organic compound (VOC) emissions during crude oil storage and transportation, this study proposes a sandwich-structured zeolite molecular sieve (SMZ) fabricated via a pressing-sintering process integrating ZSM-5 powder and granules. The resulting monolithic zeolite exhibits enhanced mechanical strength and optimized pore architecture. Systematic investigations revealed that sintering at 600 °C with 10% carboxymethyl cellulose (CMC) yielded SMZ with a specific surface area of 349.51 m^2^/g and pore volume of 0.37 cm^3^/g. Its hierarchical pore system—micropores (0.495 nm) coupled with mesopores (2–10 nm)—significantly improved adsorption kinetics. Dynamic adsorption tests demonstrated superior performance: SMZ achieved saturation capacities of 127.6 mg/g for propane and 118.2 mg/g for n-butane in liquefied petroleum gas (LPG), with a breakthrough time of 41 min and a 106% increase in adsorption capacity compared to conventional monolithic zeolite (MZ) (90.2 mg/g vs. 43.8 mg/g). Regeneration studies confirmed that combined thermal desorption (250 °C) and nitrogen purging maintained > 95% capacity retention over five cycles, attributed to the high thermal stability of the MFI topology framework (≤600 °C) and crack-resistant ceramic-like interfaces. Additionally, SMZ exhibited exceptional hydrophobicity, with a selectivity coefficient of 20.9 for propane under 60% relative humidity. This work provides theoretical and technical foundations for developing efficient and durable adsorbents for industrial VOC mitigation.

## 1. Introduction

Crude oil is a naturally occurring unrefined petroleum, composed of a complex mixture of hydrocarbons, mainly including alkanes, cycloalkanes, and aromatics, and contains a small amount of non-hydrocarbon components containing sulfur, nitrogen, and oxygen [1]. As an important energy source and industrial raw material, it is not only the basis for the production of fuels, lubricating oils, asphalt, plastics, pharmaceuticals, and agrochemical products, but also plays a key role in global energy security, petrochemical production, and geopolitical landscape [2,3,4]. However, emissions and leaks during its storage, transportation, and distribution can lead to the release of volatile organic compounds (VOCs), posing environmental risks to ecosystems and human health. These fugitive emissions not only accelerate the deterioration of oil products and the depletion of non-renewable resources, but also the leaked light hydrocarbons are more likely to form flammable and explosive mixtures with air, which will trigger major fires/explosions when encountering a fire source. Therefore, the prevention, control, and recovery of hydrocarbon emissions have urgent practical significance and are in line with the core goals of reducing greenhouse gas emissions and addressing climate change in the global sustainable development initiative [5,6,7].

The core of adsorption methods is the development of adsorbents with high specific surface area, stable physicochemical properties, and regenerability [8,9,10]. Among the many adsorption materials, zeolite molecular sieves have been widely used in adsorption, separation, and catalysis due to their rich framework structures, large specific surface area, stable chemical properties, and as materials with artificially adjustable pore structures [11,12,13]. Traditional preparation methods for zeolite molecular sieves primarily include hydrothermal synthesis, high-temperature synthesis, and vapor-phase synthesis. These methods typically yield powdered adsorbents with particle sizes ranging from tens of nanometers to tens of micrometers. Generally, powdered molecular sieves exhibit larger specific surface areas, higher pore volumes, and superior flexibility and processability, making them suitable for laboratory-scale research (e.g., adsorption isotherm and kinetic studies), high-precision gas separation (e.g., noble gas purification), and composite material development (e.g., polymer membranes doped with molecular sieves) [14,15,16]. However, powdered molecular sieves face significant limitations in industrial applications:

(1) High Pressure Drop and Mass Transfer Resistance:

Powder particles tend to form densely packed beds in fixed-bed reactors, leading to a substantial increase in pressure drop (fluid resistance). Excessive packing creates dead zones that hinder molecular diffusion, reducing mass transfer efficiency and adsorption performance.

(2) Bed Instability and Safety Hazards:

During operation, powdered molecular sieves are prone to migration or dispersion due to gas flow or vibration, resulting in bed collapse or channeling. Micron-sized fragments in fixed-bed reactors may clog downstream equipment, causing operational failures and potential safety risks. Additionally, released dust contaminates products and poses health risks to operators.

(3) High Energy Consumption for Regeneration:

Regenerating powdered adsorbents requires frequent backflushing or high-temperature treatment, which consumes significant energy. Rapid capacity decay post-regeneration necessitates frequent replenishment of adsorbents, increasing operational costs.

These challenges not only raise industrial operating costs but also restrict the application of zeolite molecular sieves in complex and large-scale scenarios. To address these issues, researchers often blend molecular sieves with binders to form monolithic adsorbents or coat them onto structured carriers. Monolithic molecular sieves exhibit continuity, structural regularity, enhanced mechanical strength, and operational stability, making them suitable for complex systems requiring long-term reliability, such as industrial catalytic reactors and continuous-flow gas separation units. However, binders act as a “double-edged sword”: while they enable shaping and provide mechanical integrity, they inevitably block molecular sieve pores, cover active sites, and degrade adsorption performance. Furthermore, organic binders may undergo side reactions with reactants at elevated temperatures, adversely affecting adsorption. Consequently, binder-free forming techniques for monolithic zeolite molecular sieves have emerged as a critical research focus [17,18,19,20,21].

Currently, preparation methods for monolithic molecular sieves mainly include solid-loading coating, compression molding, binder conversion, and 3D printing. Binder-free monolithic zeolite molecular sieves have solved some application issues of powdered and binder-formed ones [22]. However, the overall adsorption performance of the prepared monolithic zeolite may decline to some extent, depending on the preparation method and carrier used to leverage the molecular sieve’s unique advantages for different practical conditions. In this study, we put forward a method to make sandwich-structured monolithic zeolites (SMZ) by combining the benefits of powdered and granular ZSM-5 molecular sieves. The granular ZSM-5, which has good adsorption ability but is not very stable, is contained within a strong framework made of powdered ZSM-5, balancing adsorption and structure. CMC and silica sol are used as additives to adjust the pore structure and mechanical properties more accurately. Considering the complexity of crude oil volatiles with various hydrocarbon species and heteroatomic compounds, liquefied petroleum gas (LPG) is chosen as a model system for its similarity to light petroleum fractions. Dynamic adsorption experiments and advanced characterization techniques are used to analyze the structure-performance relationships and adsorption mechanisms of SMZ when interacting with volatile components. This work is of great significance to the design of binder-free monolithic zeolites and offers valuable insights into creating adsorbents for efficient hydrocarbon recovery in complex industrial settings. The methodology serves as a reference for scaling up material optimization from laboratory research to practical applications in environmental and energy technologies.

## 2. Materials and Methods

### 2.1. Fabrication of Sandwich-Structured Monolith

**Figure 1:**
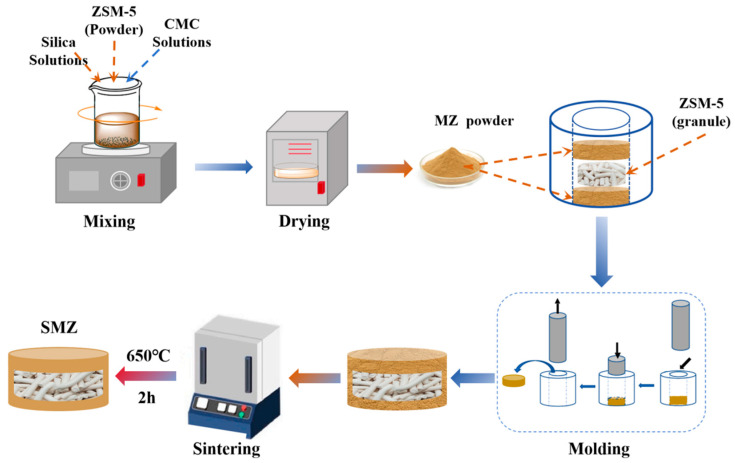
The preparation process of SMZ.

The principle of preparing monolithic molecular sieves by pressing and sintering involves combining physical pressing with high-temperature heat treatment. This method aims to provide the molecular sieve material with macroscopic mechanical strength and optimize its microspore structure, addressing industrial needs for adsorbent shaping, mechanical stability, and mass transfer efficiency. Mechanical pressure compacts the particles through van der Waals forces, hydrogen bonds, and binder bridging, forming a dense green body. Sintering then induces solid-phase reactions between the amorphous binder and the molecular sieve surface, creating a chemically bonded interface that enhances overall mechanical strength. Additionally, the thermal degradation of pore-forming agents at high temperatures generates through-pores in the monolithic zeolite, optimizing mass transfer pathways. The ZSM-5 molecular sieves used in this study were purchased from Global Molecular Sieve Co., Ltd. (Shanghai, China) and were available in both powdered and granular forms. The powdered form was used as the “shell” of the SMZ, while the granular form served as the “interlayer”. CMC served dual purposes: as a binder during shaping and as a pore-forming agent during sintering. Silica sol enhanced the plasticity of the green body and the mechanical strength of the SMZ by converting to an amorphous SiO₂ network at high temperatures, preventing pore blockage. The preparation process of SMZ is shown in Figure 1. The preparation process began by mixing ZSM-5 powder, a silica sol solution, and an aqueous CMC solution to form a uniform paste. After drying and granulation, uniformly sized precursor powder was obtained. This powder was divided into two equal portions to serve as the upper and lower parts of the “shell”, and 8 g of granular ZSM-5 was used as the “interlayer”. These components were filled into a cylindrical mold with a diameter of 5 cm in the order of “lower shell”, “interlayer”, and “upper shell” to form a layered structure. The mixture was pressed into a green body with a mass of 16 g, a diameter of 5 cm, and a thickness of 2 cm using an electric press at a pressure of 10 bar. The green body was then dried at 95 °C for 2 h and sintered in a tube furnace in air at predetermined temperatures (200 °C, 400 °C, 600 °C, 800 °C) for 2 h, followed by natural cooling to room temperature. In this study, a control group was established to prepare ZSM-5 monolithic zeolite (MZ) without a particle molecular sieve “interlayer” structure. The raw material composition of MZ is consistent with the “shell” of SMZ, and the molding, sintering methods, and final product specifications are the same as those of SMZ. In the subsequent characterization, MZ is used to represent the shell structure of SMZ.

### 2.2. Material Characterization Methods

XRD is a common characterization method for inorganic materials, used to analyze crystal structure, phase composition, and grain size. In this study, ZSM-5 was calcined in a muffle furnace at 200 °C, 400 °C, 600 °C, and 800 °C for 2 h to explore the effect of high-temperature treatment on its crystal structure and to provide theoretical guidance for the heating process of molecular sieves. The treated samples were tested using a Rigaku Smart Lab 9KW wide-angle X-ray diffractometer (Rigaku Corporation, Tokyo, Japan) with Cu Kα radiation (λ = 1.54 Å), with the following parameters: a scan rate of 2°/min, a scan range of 5–40°, a tube voltage of 40 kV, and a tube current of 30 mA. The Jade software (version 9.0) was used to compare standard PDF cards and analyze the changes in the samples’ diffraction peaks. Additionally, to further investigate the crystal structure of the SMZ and the effects of sintering temperature and additives (silica sol) on the crystal structure, XRD tests were conducted on SMZ and MZ under the same conditions. To study the macrostructure of SMZ, the surface and cross-section were observed using a FEI Nova NanoSEM 450 field-emission scanning electron microscope (Thermo Fisher Scientific, Hillsboro, OR, USA). The surface represents the shell structure (MZ), while the cross-section shows the interlayer structure (SMZ). Samples were pre-treated by vacuum sputtering with gold (10 mA, 60 s, JFC-1600 coater, JEOL Ltd., Tokyo, Japan) to enhance conductivity. Imaging parameters: acceleration voltage 5–20 kV, magnification 150–10,000×, with a focus on pore structure. Secondary electron imaging revealed microstructural features like pore distribution and macropore diameter, aiding in analyzing the differences between the shell and interlayer structures of the SMZ. The Rigaku ZSX Primus III+ spectrometer (Rigaku Corporation, Tokyo, Japan) was used to scan ZSM-5 and SMZ, recording the characteristic X-ray fluorescence intensity of each element to determine their content in the samples, particularly the silicon-aluminum ratio of the molecular sieve and the presence of impurities during preparation. The key parameters for adsorption performance characterization are the specific surface area and pore-size distribution of the adsorbent. The specific surface area reflects the effective contact sites, while the pore-size distribution indicates the selectivity towards adsorbate gases. In this study, nitrogen adsorption-desorption isotherms of SMZ and MZ were measured using a Micromeritics ASAP 2460 analyzer (Micromeritics Instrument Corporation, Norcross, GA, USA). The specific surface area was calculated via the BET method, micropore distribution was analyzed using the HK method, and mesopore characteristics were evaluated using the BJH model. Total and micropore volumes were determined by t-plot analysis. The BET equation, based on a multilayer adsorption model, describes gas adsorption on material surfaces. It considers both physical adsorption and chemisorption, assuming a uniform adsorbent surface and adsorbate gas. The equation’s expression is [23]:

(1) $$\frac{p}{V\left(P_{0}-P\right)}=\frac{1}{V_{m}C}+\frac{C-1}{V_{m}C}\cdot \frac{p}{p_{0}}$$

Among them, *P/P*_0_: Relative pressure (*P* is the actual gas pressure, *P*_0_ is the saturation vapor pressure); *V*: Adsorption volume at the current pressure (standard temperature and pressure, STP); *V_m_*: Single molecular layer saturated adsorption volume; *C*: A constant related to the adsorption heat, reflecting the interaction strength between the adsorbate and the adsorbent.

The HK equation is based on the micropore filling theory, assuming that the adsorption potential within the pores dominates the adsorption process. It is applicable to slit or cylindrical pore models. Taking the slit pore model as an example, the core equation is [23]:

(2) $$RT\ln\left(\frac{p}{p_{0}}\right)=\frac{N_{A}\varepsilon^{*}}{d-\sigma }\left[{\left(\frac{\sigma }{d}\right)}^{4}-{\left(\frac{\sigma }{d}\right)}^{10}\right]$$

Among them, *d*: Pore diameter (the distance between pore walls); *σ*: Interaction potential parameter between adsorbate molecules and pore wall atoms (such as collision diameter in the Lennard-Jones potential); ε*: Effective interaction energy parameter (related to the properties of the adsorbent and adsorbate); *R*: Gas constant; *T*: Temperature.

The BJH (Barrett-Joyner-Halenda) model is a classical method based on capillary condensation theory and the modified Kelvin equation. It is primarily used to analyze pore-size distributions in mesoporous materials (pore size 2–50 nm). The specific expression is as follows [23]:

(3) $$r_{p}=\frac{2\gamma V_{m}}{RT\ln\left(\frac{p_{0}}{p}\right)}+t\left(\frac{p}{p_{0}}\right)$$

In the BJH model, *r_p_* represents the pore radius. *γ* is the surface tension of the adsorbate in its liquid state (for nitrogen, it’s 8.88 × 10⁻^3^ N/m). *V_m_* is the molar volume of the liquid adsorbate (for nitrogen, 34.7 cm^3^/mol). *R* is the gas constant, and *T* is the temperature. *p/p*_0_ denotes the relative pressure, and *t(p/p*_0_*)* is the adsorbed layer thickness at a given pressure, often calculated using the Harkins-Jura equation.

### 2.3. Adsorption Performance Evaluation

**Figure 2:**
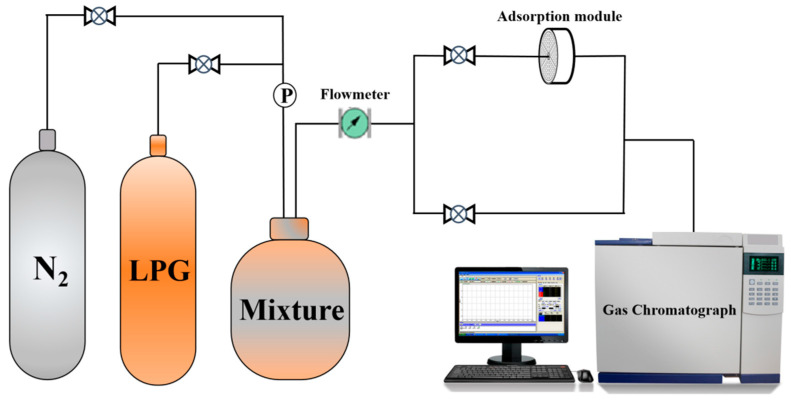
Schematic of the dynamic adsorption apparatus.

The schematic of the dynamic adsorption apparatus is shown in Figure 2. In dynamic adsorption tests, nitrogen was the carrier gas. A precision pressure-reducing valve controlled the N₂ to LPG ratio at 5:1, mixing gases into a cylinder. The mixture then passed through a flowmeter into the adsorption module, ensuring stable gas flow. This module, filled with the adsorbent, allowed alkane components in the gas to be partially adsorbed, with unadsorbed gas exiting from the other end.

A gas chromatograph (Fuli Insrtuments, Taizhou China) analyzed the hydrocarbon concentrations in the feed and exhaust gases. Regular sampling and analysis of chromatographic peaks’ intensity and area provided concentration data, yielding breakthrough curves. These curves illustrated key adsorption parameters like capacity, rate, and selectivity for different alkane components. The adsorption capacity curve and saturated adsorption capacity were determined by integrating these curves using Formula (4).

(4) $$\;\;Q=\frac{FC_{0\;}\times 10^{-6}}{m}\left(t_{s}-\int_{0}^{t_{s}}\frac{C_{t}}{C_{0}}dt\right)$$

In the formula, *Q* is the saturated adsorption capacity of VOCs (mg/g); *C*_0_, Ct are the VOCs concentrations at the inlet and outlet of the bed (mg/m^3^); m is the mass of the adsorbent (g); *t_s_* is the adsorption time, min; *F* is the total carrier gas flow rate (mL/min).

Experiments were conducted at room temperature. Before adsorption, the adsorbent bed was purged with N_2_ at 300 °C and 15 mL/min for 2 h to remove water and other impurities. Adsorption began once the bed cooled to room temperature. The inlet concentration of adsorbates was controlled at 500 ppm via dynamic dilution. Each experiment was repeated five times to ensure reliability, with data averaged after excluding outliers. The dynamic setup included gas mixing, adsorption, and data acquisition units, with quartz wool used at inlet and outlet to prevent channeling.

To gain insights into the adsorption mechanisms and behaviors of monolithic zeolites for VOCs, Freundlich, Langmuir, and Dubinin-Radushkevich (D-R) models were employed to analyze the adsorption isotherms. The Langmuir model, assuming a uniform adsorbent surface, is fitting for analyzing single-layer adsorption mechanisms, such as in microporous materials. Its mathematical expression is [24]:

(5) $$\;\;\frac{C_{e}}{q_{e}}=\frac{1}{K_{L}q_{m}}+\frac{C_{e}}{q_{e}}$$

The Freundlich model is suitable for describing multilayer adsorption on non-uniform surfaces or with diverse adsorption sites (such as mesoporous materials or physical adsorption). Its mathematical expression is [24]:

(6) $$\ln q_{e}=\ln K_{F}+\frac{1}{n}\ln C_{e}$$

The D-R model, based on the multilayer adsorption theory, is suitable for analyzing porous materials and providing adsorption energy distribution information. Its mathematical expression is [25]:

(7) $$\ln q_{e}=\ln q_{m}-K_{DR}\cdot \varepsilon^{2}$$

(8) $$\;with:\;\;\varepsilon =RT\ln\left(1+\frac{1}{C_{e}}\right)$$

In the above equations: C_e_ is the equilibrium concentration, in L/mg; *q_e_* is the maximum monolayer adsorption capacity, in mg/g; *q_m_* is the maximum monolayer adsorption capacity, in mg/g; *K_L_* is the Langmuir constant, reflecting adsorption affinity, in L/mg; *K_F_* is the adsorption capacity constant, in L/mg; 1/n is the adsorption intensity parameter (0.1 < 1/n < 1 indicates favorable adsorption); Other constants like K_DR_ are related to adsorption free energy.

## 3. Results and Discussion

### 3.1. Thermal Stability and Pore Evolution of ZSM-5

**Figure 3:**
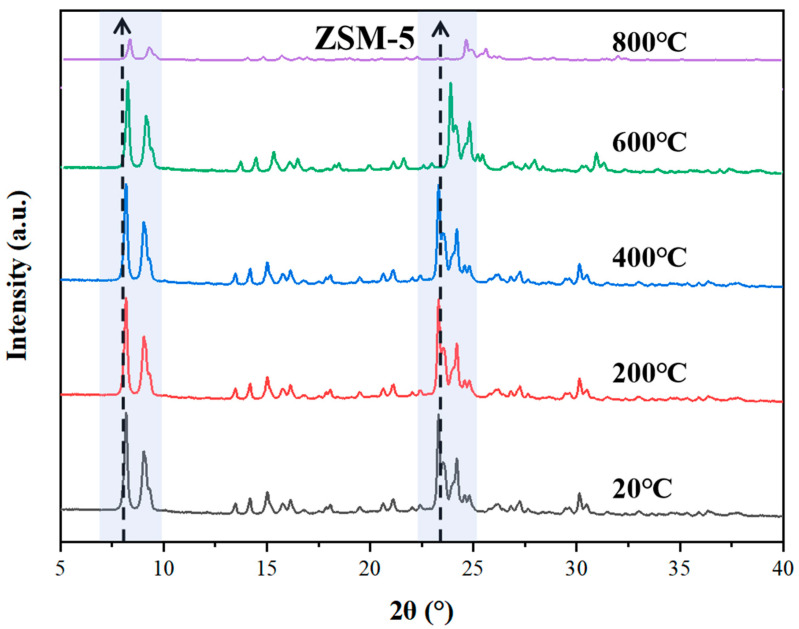
XRD Patterns of ZSM-5 at Different Temperatures.

To reveal the differential evolution laws of the crystal framework and pore structure of zeolites during high-temperature sintering, and thus select the processing method for monolithic zeolites, ZSM-5 was heat-treated in a muffle furnace at different temperatures (20 °C to 800 °C) for 2 h under an air atmosphere. The XRD patterns of the calcined zeolites are shown in Figure 3. As can be seen from the figure, the MFI-structured microporous zeolite ZSM-5 exhibits excellent high-temperature resistance. For the samples treated at temperatures ranging from 20 °C to 600 °C, the characteristic diffraction peaks were retained at 2θ = 7.9°, 8.8°, 23.1°, 23.9°, and 24.4°, corresponding to the (101), (020), (501), (303), and (133) crystal faces, respectively. The peak shifts were less than 1°, indicating that the sintering process did not significantly alter the unit cell parameters. At the high temperature of 800 °C, the intensity of each peak of ZSM-5 significantly decreased, but no amorphous signs appeared (the fluctuation of the half-peak width was less than 50%). At this point, the framework structure of ZSM-5 collapsed to a certain extent, and the microporous structure was damaged and melted. This demonstrates that the MFI framework of ZSM-5 has excellent thermal stability over a wide temperature range (≤600 °C), and some of the crystal structure can still be retained after heat treatment at 800 °C. The temperature for thermal desorption and sintering processing should be controlled around 600 °C [26].

**Figure 4:**
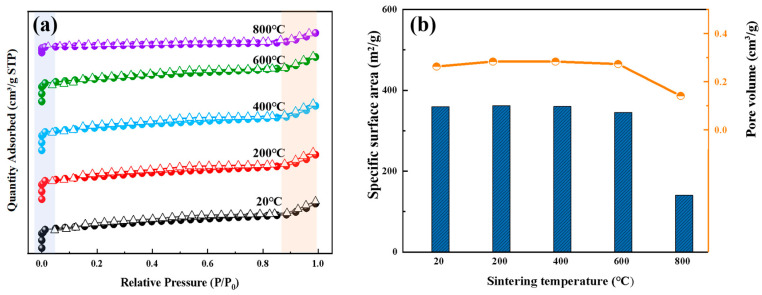
(**a**) Nitrogen adsorption desorption isotherms, (**b**) Specific surface area and pore volume of ZSM-5 zeolite at different temperatures.

This study systematically revealed the pore structure evolution of ZSM-5 molecular sieves using nitrogen adsorption-desorption isotherms and pore structure parameters. As shown in Figure 4a,b, after sintering at 200–800 °C, the specific surface area (~400 m^2^/g) and pore volume (~0.28 cm^3^/g) of ZSM-5 remained stable between 20–600 °C. The nitrogen adsorption isotherms consistently exhibited a typical Type I pattern, with a steep adsorption curve at low relative pressures (P/P₀ < 0.1), indicating a micropore-filling dominated mechanism [27]. The saturation adsorption capacity (at P/P₀ ≈ 0.9) stayed above 348 cm^3^/g, aligning with the XRD results showing crystal structure stability (peak shift < 1°). Notably, the sample treated at 600 °C showed an 8% increase in adsorption capacity in the ultralow pressure range (P/P₀ = 0.01–0.05) compared to the untreated sample. This was attributed to silanol group condensation (Si-OH→Si-O-Si), which reduced the polarity of the pore surface and weakened the interaction between nitrogen molecules and the pore walls, thereby optimizing the micropore filling efficiency. When the temperature rose to 800 °C, the microporous structure collapsed due to framework melting, causing the specific surface area and pore volume to drop to below 50% of their original values. However, the overall adsorption isotherm retained its Type I shape, indicating the structural rigidity of the MFI topology under extreme thermal conditions. Combining the XRD results, it can be concluded that the thermal stability of ZSM-5 molecular sieves can be maintained between 600 °C and 800 °C.

### 3.2. Mechanical Strength and Stability of SMZ

**Figure 5:**
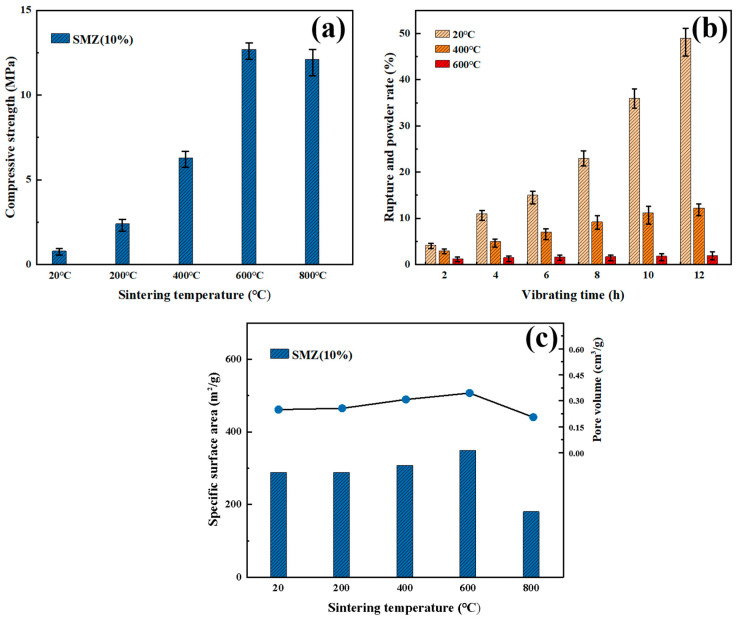
Mechanical Properties of SMZ at Different Sintering Temperatures: (**a**) Compressive Strength, (**b**) Mechanical Stability, (**c**) Specific Surface Area and Pore Volume.

Mechanical strength is crucial for the practical application of monolithic adsorbents. Figure 5a shows the compressive strength of zeolite monoliths prepared at different sintering temperatures (20 °C, 200 °C, 400 °C, 600 °C, and 800 °C) with 10% pore-forming agent addition. Results reveal a significant nonlinear relationship between sintering temperature and compressive strength. The unsintered SMZ had a compressive strength of only 0.8 MPa. When the sintering temperature increased to 400 °C, the compressive strength rose to 6.3 MPa (an increase of 687%), and the sample sintered at 600 °C reached 12.7 MPa, nearly 15 times higher than the original sample. This strengthening effect may be attributed to the reconstruction of the silicon-oxygen tetrahedron promoted by high temperatures.

Mechanical stability is also vital for the practical application of monolithic adsorbents. This study further tested the mechanical stability of SMZ (with 10% pore-forming agent addition) prepared without sintering and at sintering temperatures of 400 °C and 600 °C. The integrity of the macrostructure of the monolithic zeolite under repeated pressurization and depressurization in the pressure-swing adsorption (PSA) operation process was simulated and analyzed through a vibration table wear test, as shown in Figure 5b. The unsintered SMZ gradually disintegrated on the vibration table, with broken parts accounting for over 10% of the total mass after just 4 h of vibration. This proportion increased rapidly over time, indicating continuous structural damage under vibration. After 12 h of high-frequency vibration, the macrostructure of the unsintered SMZ was completely destroyed, with broken powder and particles accounting for 49.2% of the total mass. In contrast, the SMZ sintered at 400 °C produced only 5.6% broken material after the same 4 h of vibration, with the destruction rate slowing over time and reaching a cumulative breakage rate of 13.1% after 12 h. The sample sintered at 600 °C performed even better, with only 2.18% mass loss throughout the vibration process and no change in the macroscopic shape of the monolithic zeolite. The breakage rate dropped to below 0.5% per hour during the later stage of vibration (8–12 h), indicating effective crack propagation suppression by the ceramic-like interface formed through sintering, which strongly maintained the layered structure of the SMZ. These results show that the monolithic zeolite samples sintered at 600 °C can maintain good structural integrity in the PSA cyclic conditions simulated on the vibration table, demonstrating significantly improved mechanical stability and strong stability in practical conditions.

Figure 5c illustrates the specific surface areas and pore volumes of SMZ samples prepared at different sintering temperatures, with histograms representing specific surface areas and lines denoting pore volumes. Compared to ZSM-5 molecular sieves treated under identical conditions, the specific surface area and pore volume of SMZ samples sintered at 20 °C (unsintered) and 200 °C decreased by 16–20%. This decline is due to the incomplete thermal decomposition and phase transformation of the added pore-forming agents (carboxymethyl cellulose, CMC, and silica sol), which partially blocked the zeolitic pore structure. However, when the sintering temperature increased to 600 °C, the complete pyrolysis of pore-forming agents generated new macropores, significantly enhancing SMZ’s specific surface area (349.51 m^2^/g) and pore volume (0.37 cm^3^/g). Further increasing the temperature to 800 °C caused a drastic reduction in specific surface area (186.5 m^2^/g) and pore volume (0.22 cm^3^/g), corresponding to the collapse of the molecular sieve framework and the formation of a dense structure under extreme thermal conditions.

**Figure 6:**
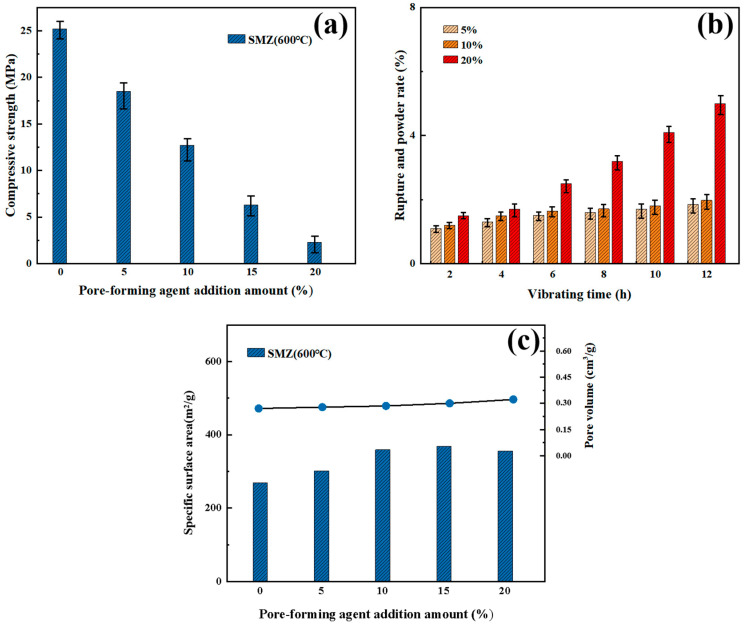
Mechanical Properties and Pore Structure of SMZ with Different CMC Additions: (**a**) Compressive Strength at 600 °C, (**b**) Mechanical Stability under Vibration, (**c**) Specific Surface Area and Pore Volume.

To balance the regulatory impact of pore-forming agent addition on the mechanical and adsorption properties of the monolithic zeolite, and to select the optimal pore-forming agent content, the compressive strength of ZSM at different carboxymethyl cellulose (CMC) addition levels was tested at 600 °C. The results are shown in Figure 6. At the sintering temperature of 600 °C, the compressive strength of SMZ without CMC addition was above 25 MPa. As the CMC addition increased from 5% to 20%, the strength gradually decreased to 2.4 MPa. This trend indicates that although the pores introduced by CMC can increase the material’s porosity, they may disrupt the continuity of the ZSM-5 microporous framework, leading to local stress concentration and a significant reduction in overall mechanical strength. Since the MFI topology of ZSM-5 relies on the close connection of silicon-oxygen tetrahedra, the introduction of pores (especially mesopores or macropores larger than micropores) weakens the effective load-bearing capacity of the crystal network, thereby reducing the overall mechanical strength.

To simulate the stability of adsorbent materials under pressure-swing adsorption (PSA) cyclic conditions, the mechanical stability of ZSM at different carboxymethyl cellulose (CMC) addition levels was further quantified through a vibration table wear test. The experimental results show that with the increase of CMC addition, the compressive strength of the adsorbent material and the structural integrity under vibration conditions both show a gradual deterioration. Referring to the specific data, the breakage rate of SMZ with 10% CMC addition was only 3.5% after 12 h of vibration, while it increased to 8.2% with 20% addition. The addition of pore-forming agents disrupts the continuity of the zeolite’s topological framework, leading to local stress concentration. The silicon-oxygen tetrahedral network of the microporous structure is highly sensitive to pore distribution, and pores with mismatched sizes weaken the load-bearing capacity of the crystal framework, thereby causing crack propagation.

Figure 6c shows the specific surface area and pore volume of SMZ with pore-forming agent addition from 0% to 20%, illustrating the pore-forming agent’s effect on the material’s pore structure. As the pore-forming agent increases, the specific surface area and pore volume rise. The sample without a pore-forming agent had a surface area of 288.2 m^2^/g and a pore volume of 0.24 cm^3^/g, while at 15% addition, these values rose to 354.2 m^2^/g and 0.37 cm^3^/g, respectively. This is due to gas release during pyrolysis, forming secondary pores and opening isolated microporous channels. However, at 20% addition, the surface area fell to 320.5 m^2^/g, while the pore volume increased to 0.38 cm^3^/g. This might be because excessive CMC leads to non-uniform pore distribution and isolated large pores, reducing local material characterization accuracy. From the trends in specific surface area and pore volume with pore-forming agent addition from 0% to 20%, it is evident that 10–15% CMC addition is sufficient for SMZ to achieve desirable specific surface area and pore volume.

In summary, the sintering temperature has a strengthening effect on the mechanical stability of the monolithic zeolite, but it can cause the collapse of the zeolite framework and the melting of the pore channels, and even the amorphization of the material. The addition of pore-forming agents (CMC) increases the specific surface area and pore volume of the material, but it systematically weakens the mechanical stability of the zeolite material by disrupting the structural uniformity of the material, reducing the thickness of the pore walls, and inducing stress concentration. Therefore, for the adsorption performance and practical application requirements (adsorption capacity vs. mechanical life), the relationship between the sintering temperature and the amount of pore-forming agent added should be adjusted to balance the mechanical stability and adsorption performance of the adsorbent material. In the following experiments on the characterization and adsorption performance of the sandwich-structured monolithic zeolite in this study, ZSM-SMZ prepared with 10% CMC addition and sintered at 600 °C was chosen as the research object.

### 3.3. Characteristics of SMZ

**Figure 7:**
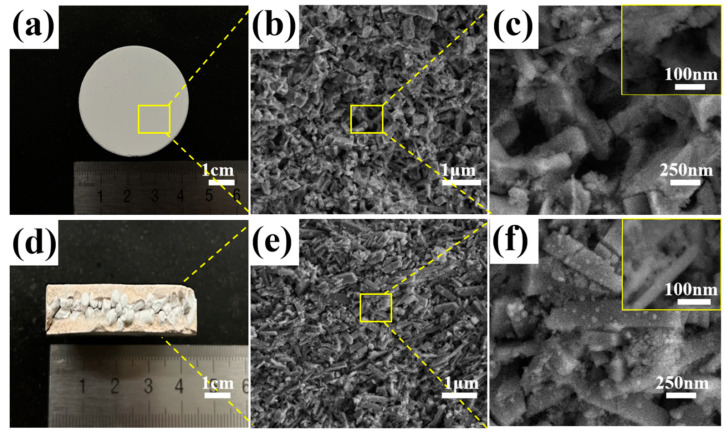
Scanning electron microscopy image of SMZ: (**a**) surface image, (**b**) surface magnified 10,000×, (**c**) surface magnified 40,000× and 100,000×, (**d**) cross-section image, (**e**) cross-section magnified 10,000×, (**f**) cross-section magnified 40,000× and 100,000×.

The optical and SEM images of SMZ are shown in Figure 7, where Figure 7a–c show the surface images of the “shell” in the SMZ sandwich structure, and Figure 7d–f show the cross-sectional images of SMZ, representing the “interlayer” in the sandwich structure and the morphology of the zeolite particles. Specifically, Figure 7a shows that the surface of SMZ is smooth and uniform, with no visible cracks or obvious pore defects. For porous adsorbent materials, internal cracks can affect the efficiency of zeolite micropores during the adsorption process; however, the controlled heating rate during the sintering process prevented the cracking of SMZ, thus avoiding the formation of defects. Figure 7d is the optical image of the cross-section of SMZ, where the sandwich structure can be clearly observed. This indicates that the original morphology of the granular zeolite was well preserved during the preparation process and was stably encapsulated in the monolithic zeolite. Figure 7b,e show the surface and cross-sectional SEM images magnified by 10,000 times, respectively. By comparison, it can be found that the surface porosity of SMZ is higher and the pore size is larger, which can be more clearly observed in the higher magnification images of Figure 7c,f. This may be attributed to two aspects of the SMZ preparation process: first, the gaps formed by the stacking of zeolite powder particles after compression, and second, the pores formed by the pyrolysis of CMC during the sintering process. A richer pore structure and higher porosity will help the adsorbent material to better exert its adsorption performance, thereby improving the pore utilization rate and mass transfer efficiency [28,29,30,31].

The elemental analysis results of ZSM-5 and SMZ are shown in Table 1. In ZSM-5, SiO_2_ accounts for 98.5687% of its total mass, while in SMZ, SiO_2_ accounts for 98.6912% of its total mass. The content of Al_2_O_3_ is 0.2924% in ZSM-5 and 0.2655% in SMZ, with silica-alumina ratios of 337 and 371, respectively. Zeolite materials with a high silica-alumina ratio exhibit greater hydrophobicity, which is crucial for treating VOCs containing moisture and other impurities under practical conditions. Additionally, the slightly higher SiO_2_ content in SMZ may be due to the addition of silica sol during the preparation process [32]. Other components such as Na_2_O and Fe_2_O_3_ are impurities in ZSM-5, with a content of less than 0.5%, and basically will not have an adverse impact on the experiment.

**Figure 8:**
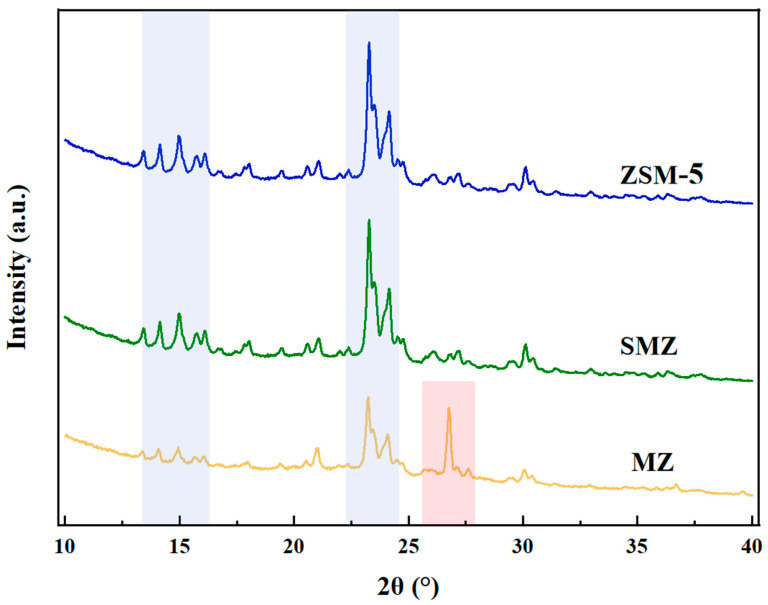
The XRD patterns of ZSM-5, SMZ, and MZ.

Figure 8 presents the XRD patterns of ZSM-5 and its different treatments (SMZ: the core of the monolithic zeolite; MZ: the shell of the monolithic zeolite), allowing for the analysis of the crystal structure of the prepared adsorbents. Based on the data obtained and using the Scherrer equation, the crystallite sizes of MZ and SMZ were estimated. The core of the SMZ (granular ZSM-5) shows characteristic diffraction peaks of the MFI topology at positions such as 2θ = 7.9° and 23.1°, with sharp and intense peaks that are highly consistent with ZSM-5, indicating the integrity of its microporous framework. Based on Scherrer equation analysis, the crystallite size of the SMZ core is estimated to be approximately 40.3 nm, confirming the preservation of the MFI framework and intact micropores. For the shell of the monolithic zeolite (ZSM-MZ), although the main peak positions did not shift significantly (Δ2θ < 0.1°), the intensity of the (501) crystal face peak (2θ = 23.1°) decreased by about 15%, and the full width at half maximum (FWHM) increased by 20%. This broadening corresponds to a reduced crystallite size of 33.5 nm, reflecting partial micropore collapse or lattice distortion induced by high-temperature sintering (600 °C), which aligns with the observed 15% decline in peak intensity. New diffraction peaks appeared in the wide-angle region of MZ (2θ > 25°), with a characteristic peak corresponding to the (101) crystal face appearing at 26.72°, which represents the quartz phase derived from silica sol added during preparation. The quartz phase exhibits a smaller crystallite size of ~27.1 nm, likely acting as a binder to enhance mechanical strength but partially blocking pore channels, thereby reducing the pore volume of MZ (0.12 cm^3^/g). In summary, the XRD analysis reveals that the sandwich-structured SMZ retains the original ZSM-5 crystallinity (40.3 nm crystallites), whereas the MZ shell suffers from sintering-induced degradation (33.5 nm crystallites) and pore obstruction by quartz nanoparticles. This structural distinction underpins SMZ’s superior specific surface area (349.51 vs. 106.25 m^2^/g) and adsorption capacity, validating its optimized pore architecture for hydrocarbon capture.

### 3.4. Specific Surface Area and Pore Structure Analysis

**Figure 9:**
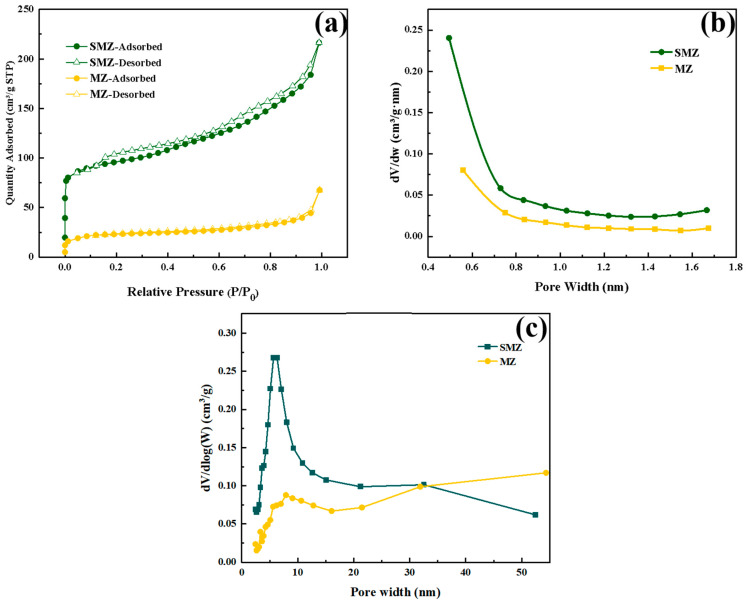
Analysis of pore structure of MZ and SMZ: (**a**) N_2_ adsorption-desorption isotherms at 298 K, (**b**) Micro pore size distribution, and (**c**) Mesoporous pore size distribution.

The specific surface area and pore-size distribution of adsorbent materials are decisive factors in determining their adsorption performance. As shown in Figure 9a, the nitrogen adsorption isotherm of SMZ rises sharply at low relative pressures (<0.1), indicating that the micropores of the material are rapidly filled with nitrogen gas. Subsequently, the isotherm exhibits a relatively flat region. As the relative pressure gradually increases to >0.8, the nitrogen adsorption isotherm of SMZ rises again with a high slope, indicating the presence of an H3 hysteresis loop. This is because capillary condensation of nitrogen gas occurs in the mesopores of the material at this point [33,34,35]. The H3 hysteresis loop is a typical feature of narrow-slit-like pores formed by cracks and aggregates of plate-like particles [36], which corresponds to the mesopores of ZSM-5 and the macroporous structure produced by particle stacking in SMZ. The nitrogen adsorption isotherm of MZ belongs to Type II isotherm, in which mesopores and macropores dominate the nitrogen adsorption, and the adsorption capacity is relatively lower than that of SMZ.

Figure 9b,c show the pore-size distribution (PSD) of SMZ and MZ based on the Saito-Foley (SF) method and the Barrett-Joyner-Helenda (BJH) method, respectively. The results show that the micropore size of SMZ is mainly distributed between 0.49 and 0.8 nm, and it has a mesoporous structure in the range of 2 nm to 10 nm. In contrast, MZ has a large number of macroporous structures distributed in the range of >50 nm, which may result from particle stacking and the pyrolysis of CMC. The final calculated results of pore size and specific surface area for SMZ and MZ are presented in Table 2. The micropore size of SMZ is 0.495 nm, which is close to the molecular kinetic diameter of most alkanes. The matching size can reduce the impact of molecular diffusion and Knudsen diffusion, increase the adsorption rate inside the pores, enhance the effective adsorption of VOCs molecules in the pores/cages, and increase the adsorption capacity of zeolite adsorbents for VOCs molecules. In addition, SMZ has a specific surface area of 349.51 m^2^/g and a pore volume of 0.28 cm^3^/g, both of which are much larger than those of MZ. A larger pore volume and specific surface area are beneficial for exposing more adsorption sites and providing a greater adsorption capacity, which are key indicators of the adsorption performance of adsorbent materials. The micropore volume of SMZ is 0.21 cm^3^/g, accounting for 70.27% of its total pore volume, and the rest belongs to the aforementioned mesopores and macropores. In practical applications, zeolites composed only of micropores provide a long diffusion path for volatile organic compound molecules, resulting in reduced adsorption efficiency of zeolites. The combination of micropores and mesopores can reduce the mass transfer resistance of VOCs inside the zeolite, allowing VOCs to quickly diffuse to the adsorption sites, thereby improving the adsorption efficiency while maintaining the selective adsorption of the material for VOCs [37].

### 3.5. Dynamic Adsorption and Isotherm Modeling

**Figure 10:**
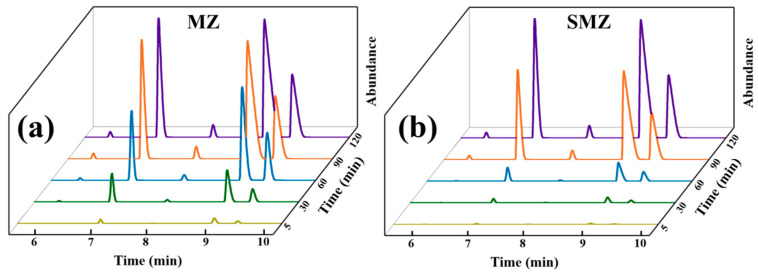
Gas chromatography (GC) spectra for MZ and SMZ in dynamic adsorption: (**a**) MZ, (**b**) SMZ.

Figure 10 shows the waterfall plot of the gas chromatogram (GC) for the LPG dynamic adsorption test. The X-axis represents the retention time of the gas chromatograph, the Y-axis is the peak intensity of the gas chromatogram, and the Z-axis is the tail-gas collection time. In the chromatogram, the distinct peaks that appear from left to right correspond to ethane, propane, propylene, isobutane, and n-butane, respectively. Along the Z-axis direction, that is, as the dynamic adsorption time is extended, the peak areas gradually increase, indicating a reduction in the available adsorption sites of the adsorbent material and a decrease in the retention capacity of the monolithic zeolite for VOCs until adsorption saturation is reached. Specifically, compared to Figure 10a, the gas chromatogram of SMZ (Figure 10b) shows a slower increase in peak area with the extension of adsorption time. The peak intensity is extremely low in the first 30 min, which means that VOCs are almost completely adsorbed by SMZ. Until the exhaust gas collected at 90 min, the concentration of VOCs accounts for half of the inlet gas. In contrast, MZ is more quickly breakthrough by VOCs and reaches saturation at 90 min, with a lower adsorption capacity. This is consistent with the aforementioned studies on the specific surface area and pore volume of MZ and SMZ. SMZ retains more microporous structures through its sandwich structure, which has practical significance for enhancing the adsorption capacity of the monolithic zeolite.

**Figure 11:**
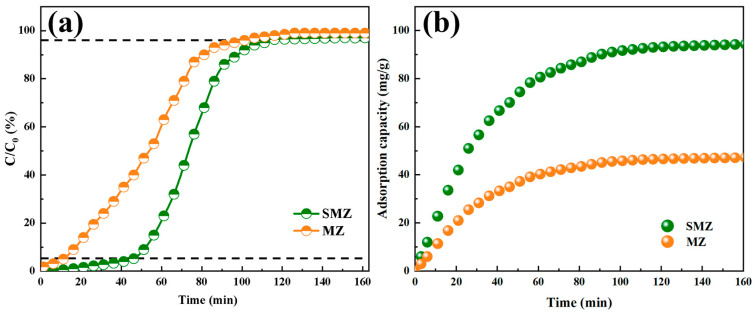
(**a**) Permeability curve of SMZ adsorbing LPG, (**b**) Adsorption capacity curve.

The dynamic adsorption results of the overall zeolite SMZ and MZ prepared by ZSM-5 for LPG are shown in Figure 11. In the permeation curve shown in Figure 11a and the adsorption capacity curve represented in Figure 11b, the permeation curve shows an S-shaped increase in the ratio of LPG concentration at the outlet to the inlet as the adsorption time increases, with the increase rate gradually increasing from slow to fast, and finally stabilizing slowly. The corresponding adsorption capacity first increases rapidly and then slowly stabilizes. Under dry conditions, the permeation times of MZ and SMZ are 16 min and 41 min, respectively, and the adsorption saturation times are 76 min and 111 min, respectively. The saturated adsorption capacities are 43.8 mg/g and 90.2 mg/g, respectively. In the dynamic adsorption experiment of LPG, SMZ has longer permeation time, saturation time, and adsorption capacity compared to MZ, which confirms the results of XRD, BET, and other tests mentioned earlier. SMZ with a sandwich structure has a larger specific surface area and pore volume, providing more adsorption sites for the overall zeolite, thereby improving the adsorption performance of the material.

**Figure 12:**
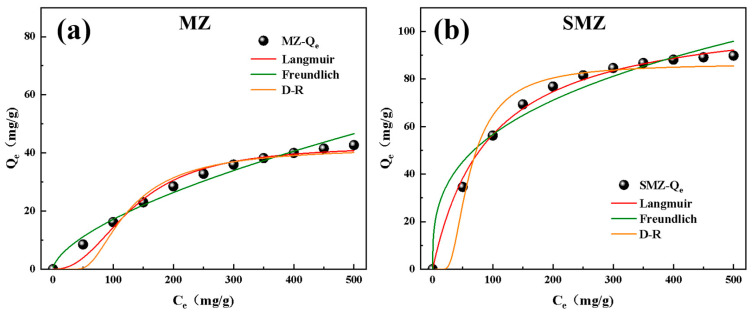
Adsorption isotherms and model fitting curves of LPG on MZ and SMZ:(**a**) MZ, (**b**) SMZ.

This study analyzed the adsorption isotherms of LPG on MZ and SMZ using the Langmuir, Freundlich, and Dubinin-Radushkevich (D-R) models. The results, shown in Figure 12a,b and summarized in Table 3, revealed distinct adsorption behaviors due to structural differences between the two materials. For MZ, the compaction and sintering process destroyed the microporous structure, increasing the average pore size to 0.553 nm, reducing the pore volume to 0.12 cm^3^/g, and forming a pore system dominated by mesopores and macropores. These factors limited its specific surface area (106.25 m^2^/g) and adsorption capacity. In contrast, SMZ, fabricated by embedding interlayers between original ZSM-5 particles and sintering with a silicasol bonding technique at 600 °C, featured a hierarchical pore system (micropores: 0.495 nm; mesopores: 2–10 nm). This structural optimization significantly enhanced its specific surface area (349.51 m^2^/g) and pore volume (0.37 cm^3^/g), thereby improving its adsorption performance. The Langmuir model indicated that SMZ had a 156% higher maximum monolayer adsorption capacity (109.2 mg/g) than MZ (42.7 mg/g) with better fitting accuracy (R^2^ = 0.98 vs. 0.93). This improvement was attributed to the match between SMZ’s micropore size (0.495 nm) and the kinetic diameter of LPG molecules. Additionally, SMZ’s theoretical maximum adsorption capacity for monolayer coverage and comparisons with other adsorbents for VOCs from literature are presented in Table 4 [38,39,40]. SMZ’s higher LPG adsorption capacity outperformed traditional molecular sieves, with its micropore-mesopore hierarchical structure adapting well to multi-component volatile petroleum gases. Furthermore, SMZ’s Langmuir adsorption equilibrium constant (K_L_ = 0.24 L/mg) was higher than MZ’s (0.073 L/mg), indicating stronger adsorption affinity or more favorable conditions, closely related to micropore optimization and mesopore-assisted mass transfer. The Freundlich model showed SMZ had a higher adsorption capacity constant (K_F_ = 52.3 mg/g·(L/mg)^1/n^) than MZ (K_F_ = 18.5 mg/g·(L/mg)^1/n^), likely due to mesopore-assisted multilayer diffusion and a hydrophobic surface modified by silicasol (SiO₂ content: 98.69%), which reduced polar site interference. The n value in the Freundlich isotherm, reflecting surface heterogeneity and adsorption intensity, was between 1 and 10 for both materials, indicating strong affinity for the adsorbate, efficient adsorption at low concentrations, and rapid uptake with increasing concentration, signifying a favorable process. The D-R model further confirmed SMZ’s micropore dominance: its adsorption free energy (E = 11.8 kJ/mol) was significantly higher than MZ’s (7.7 kJ/mol), and its lower potential constant (β = 2.5 × 10⁻^7^ mol^2^/kJ^2^ vs. 6.2 × 10⁻^7^) indicated stronger overlapping adsorption fields within micropores, enhancing efficiency. Generally, E values between 8–16 kJ/mol suggest chemisorption, while values below 8 kJ/mol indicate physisorption. Non-polar molecular sieves primarily adsorb VOCs via physical interactions, but SMZ’s micropore size closely matches VOC molecules, and its narrow micropores enhance van der Waals interactions between the adsorbate and pore walls, possibly elevating E beyond the traditional physisorption threshold while remaining within its category.

### 3.6. Desorption and Regeneration Performance

**Figure 13:**
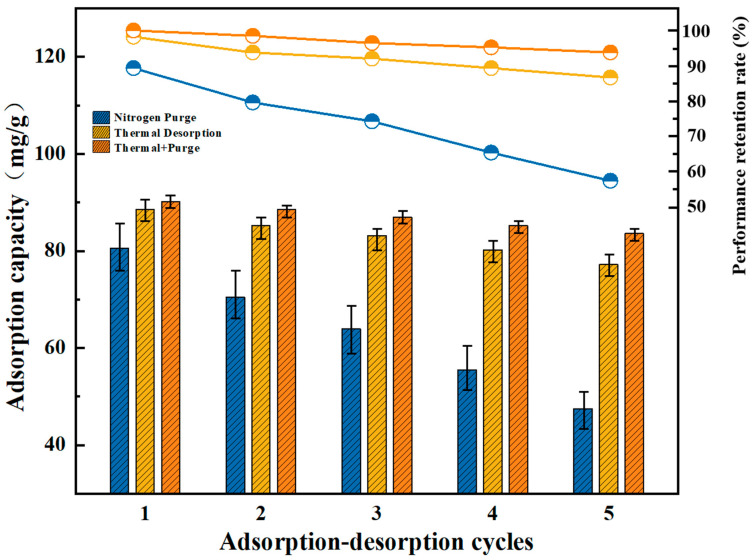
Cyclic Regeneration Performance of SMZ Under Different Desorption Methods.

The desorption and regeneration performance of adsorption materials is of significant importance for their long-term stability and economic viability in industrial applications. In this study, the desorption regeneration performance of SMZ was evaluated using inert gas purging, thermal desorption, and a combination of both methods. Nitrogen was selected as the inert gas, with a purging flow rate of 1.5 L/min for 2 h. Thermal desorption was conducted at 250 °C for 2 h, while the combined method involved 1 h of heating at 250 °C followed by 1 h of nitrogen purging. Dynamic adsorption tests under identical conditions were performed after each desorption cycle, with five consecutive adsorption-desorption cycles. The results of the three desorption methods are shown in Figure 13. When using nitrogen purging alone, the regeneration efficiency of SMZ gradually decreased from 83.5% in the first cycle to 55.6% by the fifth cycle, indicating poor desorption effectiveness. In contrast, the thermal desorption method maintained over 85% of the original adsorption capacity after five cycles. Notably, the combined thermal desorption and nitrogen purging approach preserved the material’s adsorption performance at above 95% throughout all cycles. Considering potential material wear from purge and dynamic adsorption gas flow impacts, over 95% of the adsorbent’s regeneration performance indicates nearly complete regeneration.

The desorption and regeneration performance of several common adsorption materials is summarized in Table 5. These materials, including activated carbon, silica gel, polymer resins, and MOFs, have become research hotspots due to their high specific surface areas and tunable pore structures [41,42,43,44]. However, activated carbon, silica gel, and polymer resins are limited by poor thermal and chemical stability. Their sensitivity to thermal desorption temperatures poses risks of structural ablation or even combustion during regeneration processes. In contrast, MOFs suffer from weak structural stability, leading to pore collapse or loss of active sites during desorption cycles. These limitations highlight the critical need for material design innovations to balance adsorption capacity with regeneration durability in practical applications. The high regeneration efficiency of SMZ under thermal desorption can be attributed to the ordered and stable crystal structure of ZSM-5. Its three-dimensional microporous framework (MFI topology) exhibits exceptional thermal stability at elevated temperatures. Furthermore, the monolithic zeolite, prepared via pressing and sintering, demonstrates a macroscale monolithic structure with superior mechanical strength and structural stability. These properties enable SMZ to maintain robust adsorption performance during repeated dynamic adsorption-desorption processes, potentially extending its operational lifespan significantly.

### 3.7. Adsorption Performance of Volatile Components in Crude Oil

To address VOC emissions during crude oil storage and transportation, Luo Yang’s team [45] studied the vapor-liquid equilibrium of Omani crude oil in storage tanks. Using GC-MS, they quantitatively analyzed the composition of crude oil vapors under equilibrium. At a phase equilibrium temperature of 35.00 °C, the vapor volume was 70.70 mL, with ethane (C_2_H_6_) at 0.06%, propane (C₃H₈) at 1.63%, n-butane (C_4_H_10_) at 21.56%, isobutane (C_4_H_10_) at 23.60%, and C5+ components at 40.92%. At 50.00 °C, the vapor volume increased to 139.50 mL, with ethane at 0.05%, propane at 1.62%, n-butane at 20.32%, isobutane at 23.21%, and C5+ at 42.49%. At 65.00 °C, the vapor volume further increased to 185.90 mL, with ethane at 0.05%, propane at 1.44%, n-butane at 20.17%, isobutane at 22.60%, and C5+ at 43.9%. These data show that the total volume of volatile gases in Omani crude oil vapors increases with temperature. At 35 °C, C_2_–C_4_ components (ethane, propane, n-butane, isobutane) accounted for 59.02% of total volatiles, and at 50 °C and 65 °C, they accounted for 55.20% and 56.22%, respectively. This indicates that C_2_–C_4_ components are the main fraction of Omani crude oil vapors, with a relatively stable proportion across different temperatures.

**Figure 14:**
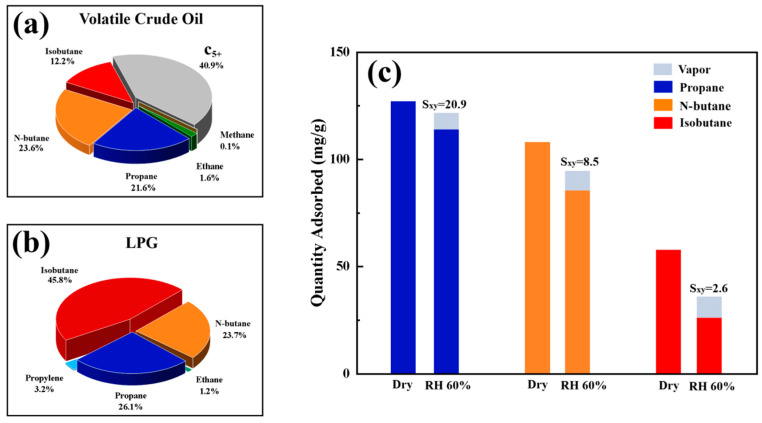
Main volatile components of crude oil and their adsorption properties: (**a**) Volatile Components Proportion of Crude Oil, (**b**) LPG Components Proportion, (**c**) Saturation Adsorption Capacity of Propane, n-Butane, and Isobutane under Dry and 60% Relative Humidity Conditions.

Figure 14a,b present pie charts comparing the volatile hydrocarbon composition of Omani crude oil from literature data and the LPG (liquefied petroleum gas) components analyzed by gas chromatography (GC) in this study. The results show that propane, n-butane, and isobutane collectively account for over 95% of the total LPG volume, aligning closely with the dominant volatile components in crude oil. This high compositional similarity validates the use of LPG depressurization to simulate the vapor emission process from crude oil storage tanks. The shared chemical properties, combined with the advantages of safety, cost-effectiveness, and operational simplicity, demonstrate the feasibility of using LPG as a surrogate for studying vapor recovery technologies targeting crude oil volatiles. Propane, n-butane, and isobutane dominate both crude oil volatiles and LPG.

Figure 14c illustrates the saturated adsorption capacities of SMZ for these three hydrocarbons and their competitive adsorption behavior with water. In single-component adsorption tests, SMZ exhibited higher capacities for smaller hydrocarbons: 127.6 mg/g for propane and 118.2 mg/g for n-butane. The pore size compatibility between SMZ and these hydrocarbons reduces molecular and Knudsen diffusion resistance, enhances intra-pore adsorption kinetics, and improves effective VOC capture within the micropores. In contrast, the saturated adsorption capacity for isobutane under dry conditions was only 58.6 mg/g, likely due to the kinetic diameter mismatch (ZSM-5 pore size: 0.49 nm vs. isobutane: 0.53 nm) and steric hindrance from its branched structure. Notably, the hierarchical pore structure (micro- and mesopores) introduced during SMZ synthesis partially mitigated these limitations, resulting in a higher isobutane adsorption capacity compared to previous studies (e.g., Xu Jianan et al. [46]). In competitive adsorption systems with water, ZSM-5-based monolithic zeolites displayed strong hydrophobicity. At 60% relative humidity (RH), the selectivity coefficients of ZSM-SMZ for propane and n-butane were 20.9 and 8.5, respectively, with adsorption capacities decreasing by 9.7% and 16.5% compared to dry conditions. The rigid siloxane network of the ZSM-5 framework creates a highly confined pore environment, effectively suppressing capillary condensation of water molecules. Additionally, its three-dimensional intersecting channels minimize the exposure of polar silanol groups (Si-OH), further reducing surface hydrophilicity. However, even minor water adsorption exacerbated the size exclusion effect for isobutane, leading to a 49.6% decline in adsorption capacity at 60% RH. To address this, future work should focus on developing micro-mesoporous composite zeolites (e.g., MCM-41/ZSM-5) to enhance isobutane adsorption under humid conditions.

Table 6 lists the maximum adsorption capacities (AC, mg/g) of common adsorbents for various VOCs to facilitate comparison. For instance, ZSM-5 has a toluene adsorption capacity of 77 mg/g, while MCM-41-ZSM-5 composite shows a high 208.9 mg/g for ethyl acetate. Industrial ZSM-5 has an 85 mg/g capacity for hexane. These data are sourced from prior studies. In our research, the monolithic zeolite exhibited a 127.6 mg/g propane adsorption capacity, indicating strong performance. Comparing adsorption capacities across different conditions allows for a comprehensive evaluation of adsorbent materials, aiding practical applications [47,48,49].

## 4. Conclusions

In summary, this study demonstrates the successful development of a sandwich-structured ZSM-5 monolithic zeolite (SMZ) with exceptional adsorption performance and industrial viability. Key findings are highlighted as follows:

(1) Adsorption Efficiency: SMZ exhibited superior adsorption capacities for propane (127.6 mg/g) and n-butane (118.2 mg/g), with a 106% improvement over conventional monolithic zeolites. Adsorption isotherm models (Langmuir, Freundlich, D-R) validated its microporous dominance and homogeneous surface interactions.
(2) Regeneration Stability: Combined thermal desorption (250 °C) and nitrogen purging achieved >95% capacity retention over five cycles, ensuring energy-efficient regeneration for industrial cyclic operations.
(3) Mechanical Durability: SMZ sintered at 600 °C showcased high compressive strength (12.7 MPa) and vibration resistance, with minimal mass loss (<2.2%) under simulated pressure-swing adsorption (PSA) conditions, guaranteeing long-term structural robustness.
(4) Pore Engineering: The hierarchical pore architecture (0.495 nm micropores + 2–10 nm mesopores) optimized mass transfer, enabling rapid VOC capture even under 60% humidity, while binder-free fabrication eliminated pore-blocking risks.

These results position SMZ as a scalable, cost-effective solution for VOC recovery in oil storage and petrochemical industries. Future work should focus on micro-mesoporous composites (e.g., MCM-41/ZSM-5) to enhance branched alkane adsorption and refine sintering protocols for humid environments. This study bridges laboratory innovation to industrial application, offering a sustainable pathway for mitigating hydrocarbon emissions.

## Figures and Tables

**Table 1:** Elemental composition of ZSM-5 and SMZ.

Component	Content (%)	
	ZSM-5	SMZ
SiO_2_	98.5687	98.6912
Na_2_O	0.4465	0.3865
Al_2_O_3_	0.2924	0.2655
Fe_2_O_3_	0.1842	0.1632
P_2_O_5_	0.1355	0.1165
K_2_O	0.1326	0.1218
CaO	0.0438	0.0324
MgO	0.0230	0.0152
Other	0.1733	0.2077

**Table 2:** Textural properties of SMZ sample.

Sample	S_BET_ (m^2^/g)	V_total_ (cm^3^/g)	V_micro_ (cm^3^/g)	Average Pore Size (nm)	Micropore Size (nm)
MZ	106.25	0.12	0.05	5.27	0.553
SMZ	349.51	0.37	0.26	4.19	0.495

**Table 3:** Fitting parameters of adsorption isotherm models.

Theoretical Model	Parameter	MZ	SMZ
Langmuir model	q_e_/(mg·g^−1^)	42.7	109.2
	K_L_/(L·mg^−1^)	0.073	0.24
	R^2^	0.93	0.98
Freundlich model	K_F_/(L·mg^−1^)	18.5	52.3
	n	1.8	2.2
	R^2^	0.96	0.93
D-R model	q_e_/(mg·g^−1^)	42.1	88.4
	β/(mol^2^·kJ^−2^)	6.2 × 10^−7^	2.5 × 10^−7^
	E/(kJ/mol)	7.7	11.8
	R^2^	0.95	0.97

**Table 4:** Langmuir monolayer adsorption capacities (q_e_) for VOCs.

Materials	Adsorbate	q_e_/(mg·g^−1^)	References
Activated biochar	Phenol	106.2	[38]
ZSM-5	propane	101.6	[39]
4A molecular sieve	Ethane	78.3	[40]
SMZ	LPG	109.2	This study

**Table 5:** Desorption and Regeneration Performance of Common Adsorption Materials.

Materials	Adsorbate	Desorption Method	Recovery Rate	References
Activated Carbon	Benzene	Electro-thermal	79% (4 times)	[41]
Silica Gel	Ammonia	Vacuum + purging	81% (3 times)	[42]
Polymer Resin	n-Hexane	Thermal	32% (4 times)	[43]
MOF	n-Hexane	Radiation	83% (3 times)	[44]
SMZ	LPG	Thermal + Purging	96% (5 times)	This study

**Table 6:** Adsorption Capacity Comparison of ZSM-5-Based Materials for VOCs.

Materials	Adsorbate	AC (mg/g)	References
ZSM-5	Toluene	77	[47]
MCM-41-ZSM-5	Ethyl acetate	208.9	[48]
Industrial ZSM-5	Hexane	85	[49]
Monolithic Zeolite	propane	127.6	This study

## Data Availability

Data are contained within the article.
